# Submergence Response and Tolerance of Rice: Morphological and Physiological Strategies, and Regulatory Mechanisms

**DOI:** 10.3390/antiox15101253

**Published:** 2026-09-30

**Authors:** Tingting Feng, Shengqin Liu, Boxin Shi, Liangliang Lv, Xinyu Huang, Ruining Li, Hao Ai, Zhanglun Sun, Aifeng Zhou, Yali Liu, Rongfeng Cui, Yuan Liu, Xianzhong Huang

**Affiliations:** 1 Bio-Breeding Laboratory of Anhui Province, College of Agriculture, Anhui Science and Technology University, Chuzhou 233100, China; 11655@ahstu.edu.cn (T.F.); liusq727826@163.com (S.L.); sbx18326495144@163.com (B.S.); 18693250227@163.com (L.L.); h1848511758@163.com (X.H.); lirn@ahstu.edu.cn (R.L.); aihao@ahstu.edu.cn (H.A.); sunzl127425@126.com (Z.S.); 2 College of Life Sciences, Shihezi University, Shihezi 832003, China; 3 Anhui Xin Fu Xiang Tian Ecological Agriculture Co., Ltd., Ma’anshan 238200, China; xinfuxiangtian@126.com; 4 Institute of Agricultural Economics and Development, Chinese Academy of Agricultural Sciences, Beijing 100081, China; liuyali@caas.cn; 5 Beijing Academy of Forestry and Landscape Architecture, Beijing 100102, China; cuirongfeng@163.com; 6 Biotechnology Research Institute, Chinese Academy of Agricultural Sciences, Beijing 100081, China

**Keywords:** rice, submergence, hypoxia and anoxia, ROS, phytohormonal crosstalk, breeding

## Abstract

Rice, as a globally vital food crop, is frequently threatened by flooding, which adversely affects its growth and yield. The most immediate and prominent influence of waterlogging or submergence induced by flooding on rice is hypoxia (reduced oxygen availability) or anoxia (absence of oxygen) stress and the consequent energy deficiency. Rice has evolved specific acclimation strategies to counteract or alleviate flooding stress, including leaf gas film (LGF) formation, adventitious root (AR) emergence, aerenchyma formation, radial oxygen loss (ROL) barrier development, and the stunted or elongated growth of coleoptiles or internodes, all of which serve to secure oxygen supply and maintain energy homeostasis for sustaining survival. These adaptive traits and strategies are governed by the regulatory genes involved in the reprogramming of energy and carbohydrate metabolism, the accumulation and scavenging of reactive oxygen species (ROS), and the programmed cell death (PCD) process, as well as the phytohormone biosynthesis and signaling pathways. To facilitate a comprehensive understanding of the submergence response and tolerance and the intricate regulatory mechanisms in rice, this review integrates the impacts of flooding on the growth and development, the strategies employed to adapt to submergence, the physiological and biochemical characteristics, the phytohormonal signaling and crosstalk, and the molecular mechanistic framework of rice subjected to submergence stress. It also highlights the importance of key superior genes for flooding-tolerance, flooding-tolerant germplasms, and molecular breeding technologies in developing submergence-tolerant rice varieties. By aligning these advances, this review provides valuable insights into and guidance for improving resilience to submergence stress and enhancing crop production under constantly changing climate conditions.

## 1. Introduction

Flooding not only directly destroys farmland infrastructure; more importantly, the associated submergence stress is a major factor leading to substantial yield losses or even complete crop failure [1,2]. According to statistics from the Global Disaster Data Platform (https://www.gddat.cn), 842 major flood events occurred worldwide between 2020 and 2024, affecting up to 200 million people and causing economic losses of approximately USD 220 billion, with agricultural production suffering devastating damage. As the staple food for more than half of the world’s population, rice is predominantly cultivated in the monsoon and tropical regions of East Asia, Southeast Asia, and South Asia. These regions are frequently affected by flooding, and the resulting submergence stress poses a serious threat to rice production and global food security [3,4].

**Figure 1:**
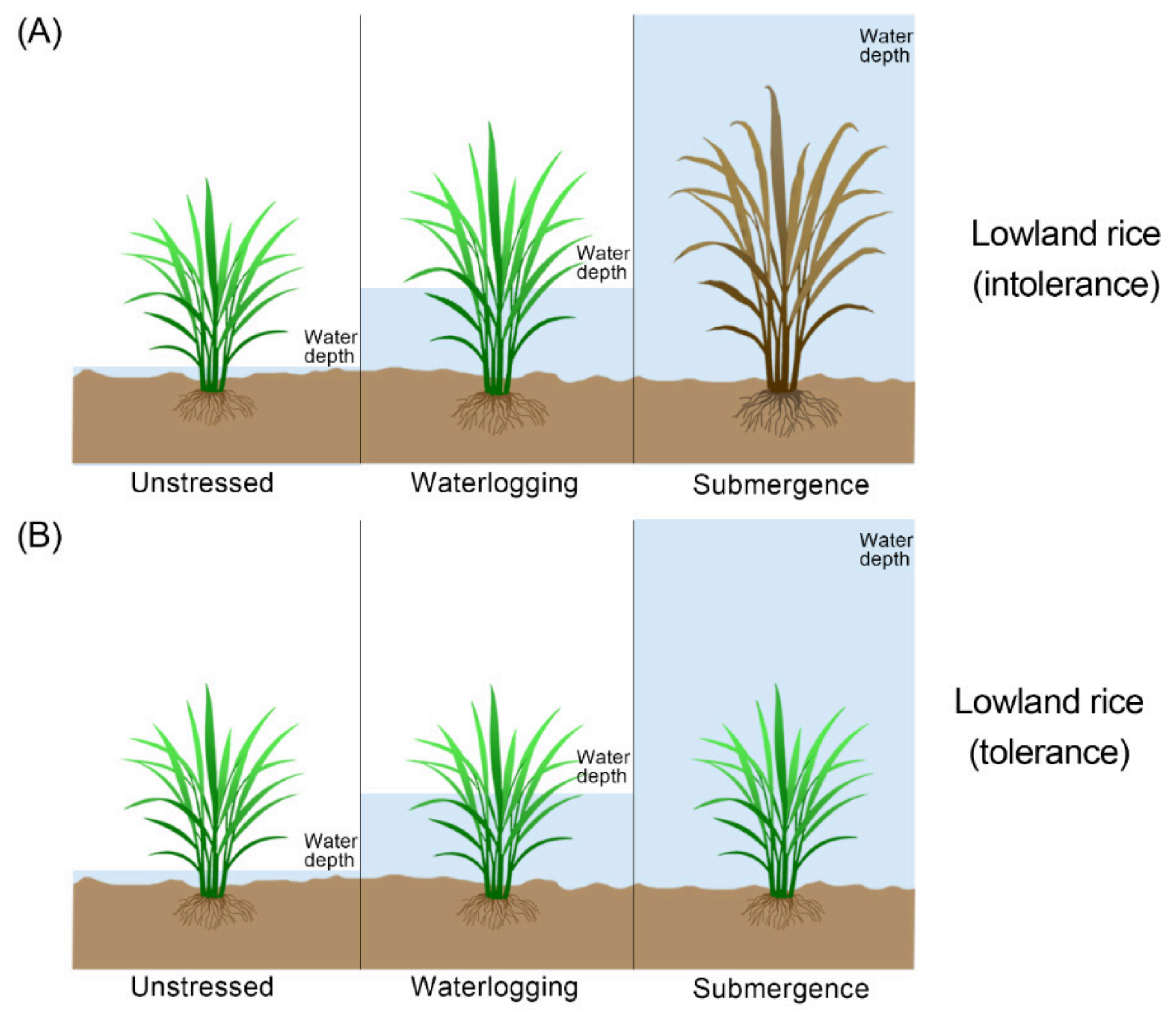

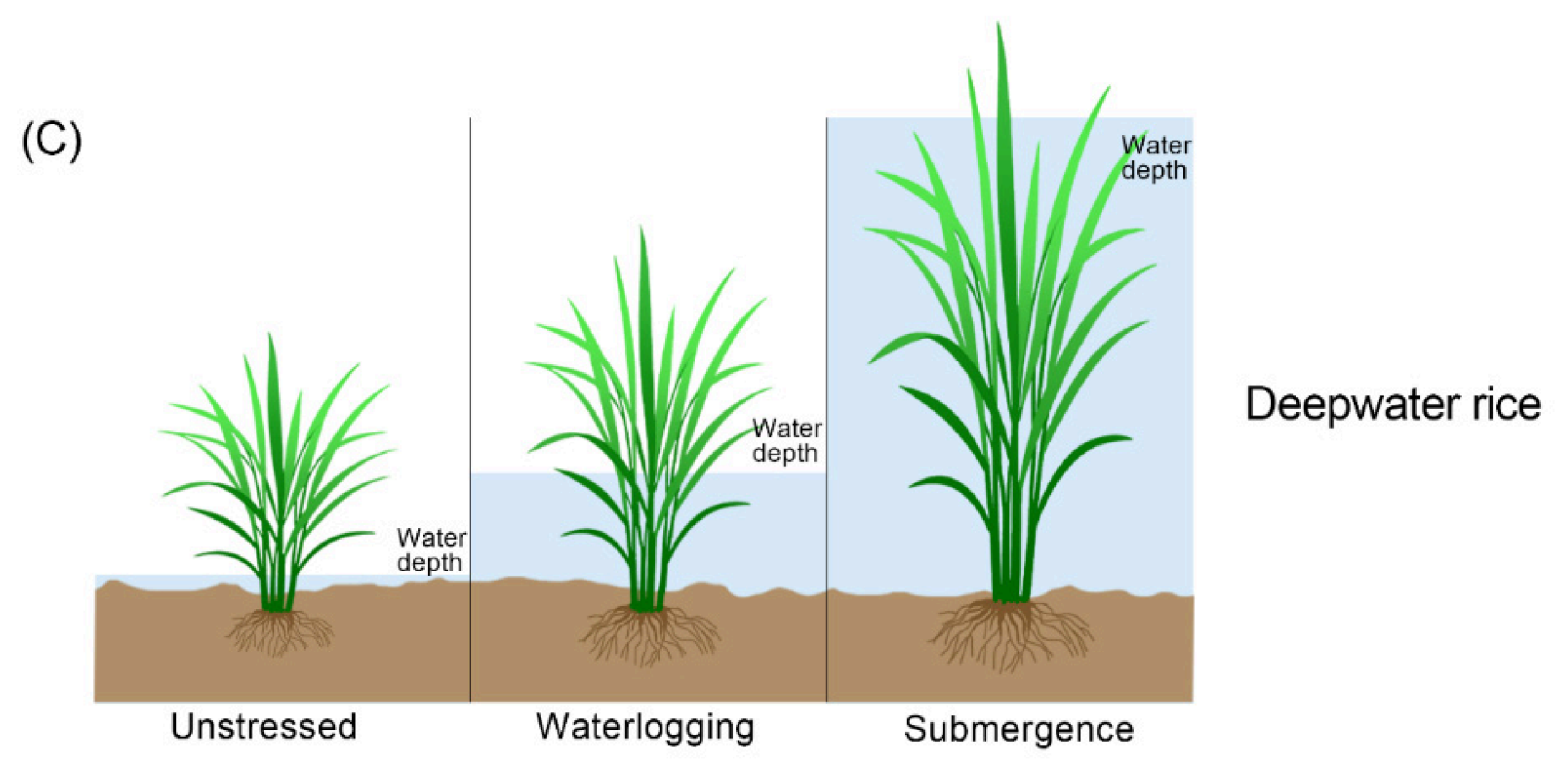
Different rice varieties exhibit distinct adaptive characteristics and responsive strategies under waterlogging and submergence conditions. (**A**) The responsive strategy of intolerant lowland rice under waterlogging and submergence conditions. (**B**) The responsive strategy of tolerant lowland rice under waterlogging and submergence conditions. (**C**) The responsive strategy of deepwater rice under waterlogging and submergence conditions.

Submergence-related stress can generally be classified into two types: waterlogging and submergence (Figure 1) [5,6]. Waterlogging is usually caused by prolonged rainfall or river flooding, which fills soil pores with water and exposes the underground parts of plants, particularly the roots, to hypoxic or anoxic conditions. Under waterlogging conditions, the aboveground parts of plants are partially or not submerged. However, plants suffer severe damage and injury due to the oxygen deprivation, impaired root function, reduced nutrient uptake, exacerbated microorganism growth and accumulated toxic substances [7,8]. By contrast, submergence generally refers to the immersion of the entire or a significant portion of a plant body in water. Submergence-induced low light availability, hypoxia or anoxia, and reduced carbon dioxide concentrations severely restrict photosynthesis and cellular respiration, and cause enormous metabolism changes. Furthermore, when floodwaters recede and normal light and oxygen conditions are restored, plants are subjected to multiple stresses including secondary oxidative stress, dehydration, photoinhibition, and accelerated senescence, ultimately causing injury and death [9,10,11,12].

To withstand and address the challenges posed by the intensification of global climate change and ensure food security, it has become a key scientific problem and technical bottleneck to clarify the genetic mechanisms that regulate crop responses to submergence-related stress and adaptation to submergence, and to formulate corresponding breeding and acclimatization strategies to deal with these agricultural threats. Therefore, a comprehensive review of the morphological, physiological, biochemical, and molecular regulatory responses of rice to flooding stress will contribute to deepening the understanding of the mechanisms underlying the submergence response in crops, and provide valuable insights into alleviating submergence stress and improving tolerance and resistance to flooding for crops, as well as increasing their productivity in flood-prone regions.

## 2. Effects of Flooding on Rice Growth and Development

The impacts of flooding disasters on rice growth and development span the entire life cycle of the plant [3,13,14]. During germination and the early seedling stages, excessive water causes oxygen deficiency around seeds, which suppresses aerobic respiration and forces the rapid elongation of the coleoptile and seedling to reach the water surface [8,15]. This process consumes large amounts of carbohydrate reserves, resulting in an insufficient energy supply and a sharp decline in the germination rate and seedling survival [8,16]. In addition, O_2_ deprivation inhibits mitochondrial oxidative phosphorylation. Consequently, ATP production and NAD^+^ regeneration depend on inefficient glycolysis and fermentation, which leads to the emergence of the toxic by-product acetaldehyde, cytoplasmic acidification, and cell death [8,12,17,18].

After entering the tillering stage, hypoxic conditions restrict the efficiency of rice roots in absorbing and transporting nutrients such as nitrogen, and reduce photosynthesis and assimilate accumulation [19,20,21,22,23,24]. Consequently, rice plants exhibit reduced tiller number, yellowing and senescence of leaves, and inhibited biomass accumulation [3,11,15,19,21,25]. Moreover, this inhibitory effect significantly intensifies as the depth of the flood increases [3].

During the reproductive growth stage, especially the panicle initiation and grain-filling stages, rice becomes significantly sensitive to flooding [3,13]. Panicle initiation is a critical period for floral organ differentiation and development, and submergence may inhibit the normal formation of spikelets at this stage, resulting in a reduced number of spikelets per panicle [3,13]. During grain filling and maturation, grain development depends on the continuous supply of photosynthates from leaves and efficient phloem transport [26]. Flooding stress accelerates the senescence of functional leaves, blocks the formation and growth of new leaves, decreases the leaf area index, and reduces the supply of assimilation products to grains, ultimately resulting in decreased grain weight, substantial yield losses and deteriorated grain quality [3,13,19,21,27].

## 3. Morphological and Structural Defenses of Rice Under Submergence Stress

**Figure 2:**
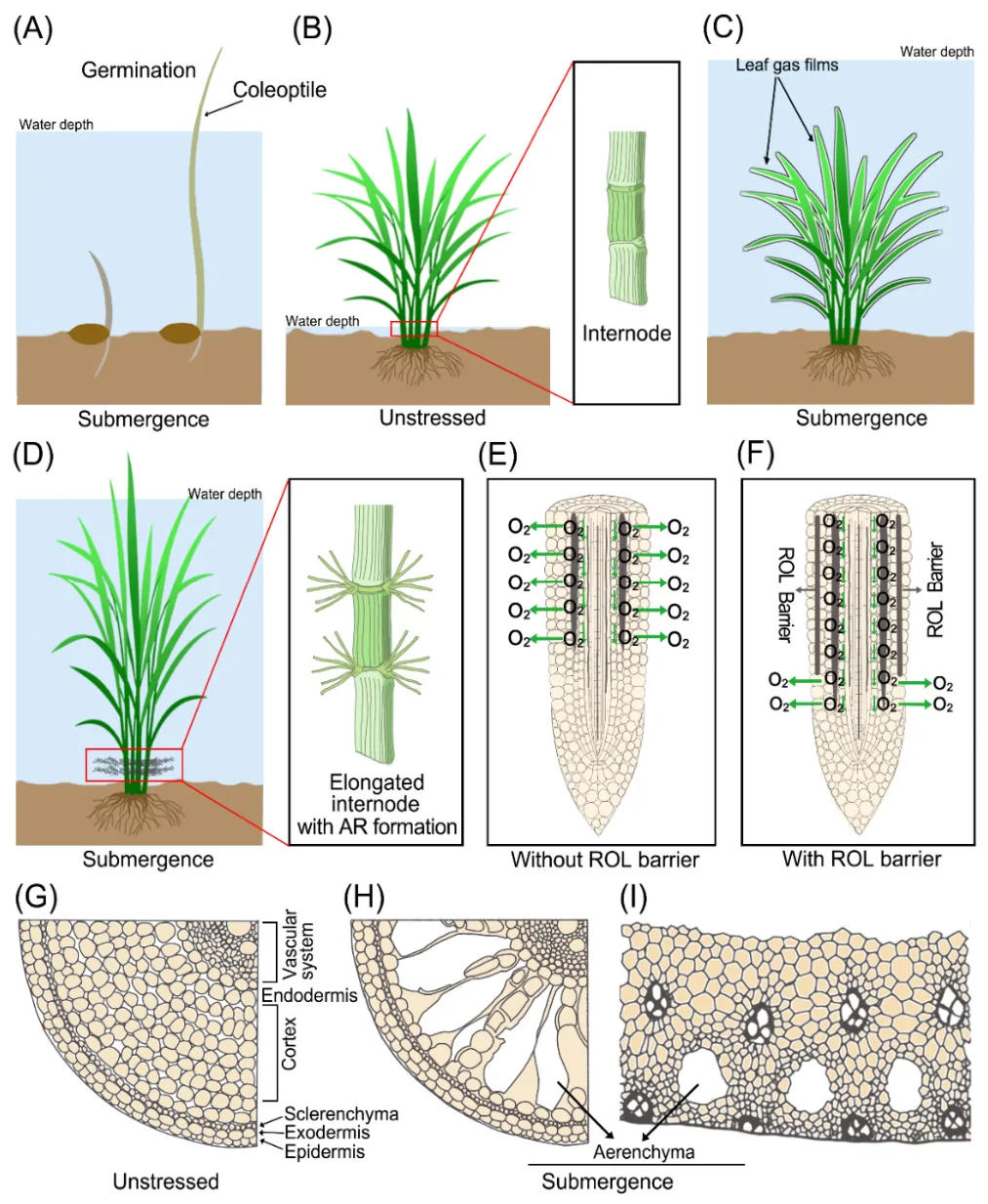
Morphological and anatomical adaptations of rice under submergence stress. (**A**) Schematic diagram of the elongated coleoptile during seed anaerobic germination under submerged conditions. (**B**) Schematic diagram of rice with the unelongated internode under the unstressed conditions. (**C**) Formation of leaf gas film (LGF) under submergence stress. (**D**) Schematic diagram of rice with the elongated internode and adventitious roots (ARs) under submergence stress. (**E**,**F**) Schematic diagrams of rice roots without (**E**) and with (**F**) the radial oxygen loss (ROL) barrier. (**G**–**I**) Schematic diagrams of root structure under unstressed conditions (**G**) and formation of aerenchyma in roots (**H**) and stems (**I**) under submergence stress.

Plant species exhibit substantial morphological and anatomical variations in their tolerance to flooding [28,29]. Many dryland crops, such as wheat, are sensitive to anaerobic conditions and detrimentally affected by submergence. Therefore, they are classified as flooding-sensitive species [18,28,29,30,31]. Some grasses and trees can endure and tolerate prolonged flooding, and are classified as flood-tolerant species [32,33,34]. In contrast, rice can grow under waterlogged conditions and exhibits a certain degree of resistance to submergence stress, and is thus classified as a wetland plant [35]. The submergence tolerance of rice mainly depends on a series of structural defense mechanisms, including leaf gas film (LGF), adventitious root (AR), aerenchyma, and the radial oxygen loss (ROL) barrier (Figure 2). These structures improve internal oxygen supply, reduce oxygen loss, and establish alternative absorption and support systems, thereby alleviating and mitigating hypoxic or anoxic stress in aerial parts, roots and surrounding soils [6,22,30]. In addition, the elongation of the coleoptile during the germination stage and the elongation of internodes during the vegetative or adult stage will be discussed in Section 7.4.

### 3.1. LGF: “Micro Air Chamber” Underwater

Rice leaves possess wax-coated and superhydrophobic surfaces. When submerged, a stable and thin air layer is maintained on the leaf surface, referred to as the LGF (Figure 2C). This gas film effectively separates the leaf surface from the surrounding water by forming an interface between the gas and water phases. The LGF provides a relatively stable source of CO_2_ for photosynthesis and O_2_ for respiration, thereby significantly alleviating hypoxia or anoxia underwater [30,36,37,38]. However, the gas film gradually becomes thinner with time of water immersion and eventually disappears within a few days, leading to reduced photosynthesis and oxygen supply [39,40].

Epicuticular wax, the outermost component of the leaf epidermis, plays a crucial role in leaf hydrophobicity and gas film retention capacity [30,41]. Cuticular wax biosynthesis involves fatty acid elongation as well as the production of alcohols, esters, aldehydes, and alkanes [40,41]. In rice, *LGF1*/*OsHSD1* encodes a hydroxysteroid dehydrogenase with NAD^+^/NADP^+^-dependent activity that can utilize mammalian steroid substrates in vitro and regulate C30 primary alcohol synthesis in vivo, thereby affecting wax platelet abundance, surface hydrophobicity, gas film retention, and photosynthesis under submerged conditions [30,40,42]. A loss-of-function mutant of *LGF1*/*OsHSD1*, *dripping wet leaf 7*/*oshsd1*, exhibits increased contents of very-long-chain fatty acids and soluble fatty acids, decreased C30 primary alcohol, reduced epicuticular wax crystals, and thicker cuticle membrane, and quickly loses its gas films underwater. These changes caused a significant reduction in net photosynthesis under submergence conditions [40,42].

### 3.2. Formation and Structural Adaptations of ARs

Prolonged submergence and hypoxic/anoxic conditions can cause the growth inhibition or even the gradual death of pre-existing root systems due to the inhibition of aerobic respiration [29,43]. In response, rice activates AR primordia at the stem nodes, which subsequently penetrate the epidermis and develop into aerial or floating adventitious roots (Figure 2D). These newly formed roots typically exhibit increased aerenchyma development and reduced radial oxygen loss rates. This enables efficient oxygen uptake from the atmosphere and transport to underground tissues, and minimizes the distance for its diffusion, thereby sustaining essential physiological functions [43,44,45,46].

The AR formation in rice is a highly regulated developmental process involving complex molecular signaling networks, hormonal interactions, and environmental cues. Under aerobic conditions, auxin accumulates at specific sites in the nodes or the hypocotyl through polar transport, thereby initiating root primordium formation [18,47]. Under hypoxic or anoxic conditions, root primordia embedded under the epidermal layers of nodal tissues are induced by ethylene (ETH) to emerge [48]. The growing primordium exerts a mechanical force directed on the overlying epidermal cells, which provides spatial resolution and is necessary for reactive oxygen species (ROS)-mediated epidermal programmed cell death (PCD) in addition to ETH signaling [43,49,50]. Moreover, ETH interacts intricately with other phytohormones. For instance, gibberellic acid (GA) acts synergistically with ETH to promote AR emergence and elongation, whereas abscisic acid (ABA) suppresses this process. Together, these hormones form a dynamic regulatory network that fine-tunes adventitious root development [43,49,51,52].

### 3.3. Aerenchyma: “Green Oxygen Channel” Underwater

Aerenchymas are the continuous gas-filled spaces that are widely present in the roots and stems of many plant species [43,53,54,55]. They enable the efficient internal transport of oxygen in the aerial parts of plants to the roots and rhizosphere, thereby maintaining root respiration and viability. They also facilitate the removal of the soil- or root-derived gases (e.g., ETH, CO_2_, and methane) to the shoots and atmosphere. They are therefore essential for plant survival under flooding and other hypoxic stresses [43,46,54,56,57].

Aerenchyma is formed by PCD and subsequent lysis of specific cell types, such as parenchyma cells in shoots or cortical cells in roots (Figure 2G–I). According to their formation patterns, the aerenchyma in rice can be classified into constitutive and inducible types. Constitutive aerenchyma forms in the roots of rice plants under well-drained aerobic conditions and the process is regulated by auxin/indole-3-acetic acid protein (AUX/IAA)- and auxin response factor (ARF)-dependent auxin signaling pathways. The presence of constitutive aerenchyma enables roots to maintain a partial oxygen supply during early flooding and allows for the rapid induction of additional aerenchyma formation thereafter [54,56,57].

The formation of inducible aerenchyma represents an efficient adaptive mechanism to hypoxic/anoxic stress, and is a key trait that enables rice to survive frequent flooding. Under waterlogging conditions, restricted gas exchange leads to ETH accumulation in the roots. Hypoxia also induces the expression of genes encoding key ETH biosynthesis enzymes, including 1-aminocyclopropane-1-carboxylic acid (ACC) synthase (ACS) and ACC oxidase (ACO), which further enhances ETH accumulation. Elevated ETH stimulates the expression of a gene encoding the respiratory burst oxidase homolog (RBOH) isoform H (RBOHH), which is activated by Ca^2+^-dependent protein kinases (CDPKs) following an influx of cytosolic Ca^2+^, resulting in ROS production. Additionally, the reduced expression of *METALLOTHIONEIN1* (*MT1*) genes, which encode ROS scavengers, may promote the accumulation of ROS in the rice cortex. ROS subsequently trigger the induction of PCD in cortical parenchyma cells, leading to cavity formation and aerenchyma development [29,57,58,59]. Application of ETH or suppression of ROS-scavenging systems, such as downregulation of *METALLOTHIONEIN2b* (*MT2b*), further increases ROS levels and accelerates aerenchyma formation in rice internode parenchyma cells [29,55]. Conversely, application of inhibitors of RBOH activity or cytosolic calcium influx suppresses ROS accumulation. It blocks inducible aerenchyma formation, thereby demonstrating the essential role of the ETH-ROS signaling pathway in regulating inducible aerenchyma development [57,58].

### 3.4. ROL Barrier: “Outer Apoplastic Barrier”

Under submerged conditions, the oxygen transported longitudinally through the aerenchyma from the shoots towards the root tips is prone to diffusing radially into the rhizosphere and soil, a process known as ROL [53,60]. To diminish loss of O_2_ to the surrounding environment, plant roots induce the formation of an ROL barrier that maintains oxygen supply to the root apex while blocking the entry of gases and toxic soil compounds (i.e., methane, hydrogen sulfide and reduced metal ions) as well as pathogens. This improves root respiration and nutrient uptake, and enables normal root growth in anaerobic soils (Figure 2E,F) [46,60,61,62].

The ROL barrier is mainly formed by suberin and lignin deposition in the exodermis and/or sclerenchyma cell walls [45,62,63]. In addition, glycerol esters are key sealing components that are deposited in suberized exodermal cell walls. Together with suberin and lignin, they form a dense composite barrier structure. Studies have shown that *LGF1* expression is closely associated with barrier function. Flooding stress strongly induces *LGF1* expression in roots, particularly in the outer 10–20 mm region behind the root apex, where functional biopolymers are deposited during the formation of the outer apoplastic barrier. Under flooded conditions, loss-of-function mutants of *LGF1* exhibit significantly reduced glycerol ester accumulation in the outer parts of root tissues, resulting in a weaker ROL barrier, reduced internal oxygen retention capacity, and a significantly higher rate of radial oxygen loss compared with wild-type plants [64].

## 4. Reprogramming of Energy Metabolism

Under normal aerobic conditions, rice efficiently generates adenosine triphosphate (ATP) through mitochondrial aerobic respiration, sustaining cellular functions and growth. However, when rice is subjected to submergence stress, the rapid decline in dissolved oxygen levels can expose its roots and submerged tissues to hypoxia or anoxia. This leads to the suppression of the mitochondrial respiratory chain and a sharp reduction in the efficiency with which ATP is produced [12,65]. This energy crisis directly inhibits seed germination, seedling emergence, vegetative growth, grain filling, and ultimately reduces field emergence rates and yield.

To cope with energy deficiency under hypoxic/anoxic conditions, rice rapidly activates less efficient anaerobic metabolic pathways in order to generate the minimal amount of energy required for survival. During hypoxic/anoxic stress, the expression of specific α-amylase genes is activated, catalyzing the hydrolysis of starch into glucose to maintain substrate availability for glycolysis [17,18,66,67]. Concurrently, sucrose is rapidly degraded into glucose and fructose via the energy-saving sucrose synthase (SUS) pathway, ensuring that carbon flux is preferentially directed towards energy production rather than storage [17,67,68]. Hypoxic stress also induces the high expression of *OsHXK7* encoding hexokinase, which senses and phosphorylates glucose, thereby facilitating its entry into the glycolytic pathway and improving fermentation activity [69]. However, glycolysis produces large amounts of pyruvate. Under impaired mitochondrial function, pyruvate is further metabolized into lactate or ethanol through the action of key enzymes such as lactate dehydrogenase (LDH) and alcohol dehydrogenase (ADH), concomitantly regenerating NAD^+^ to sustain glycolytic flux. This fermentative metabolism yields only 2 to 4 mol ATP per mol hexose, which is far lower than the energy output of aerobic respiration. Moreover, fermentation rapidly depletes carbohydrate reserves and disrupts cytosolic potential of hydrogen (pH) homeostasis, leading to cytosol acidification and injured membrane integrity [17,18,66,67]. Consequently, although anaerobic metabolism can temporarily support survival, its low efficiency and high carbon cost combined with the restrained photosynthesis result in the rapid exhaustion of carbohydrate reserves and insufficient energy supply, and ultimately endanger the long-term survival of plants under prolonged O_2_ deprivation or deficiency.

To adapt to the energy limitations imposed by short/long-term submergence, rice has evolved two distinct acclimation and survival strategies through prolonged natural selection: the quiescence strategy and the escape strategy [70,71]. The quiescence strategy, also known as low-oxygen quiescence syndrome (LOQS), is characterized by stunted growth and repressed carbohydrate consumption, enabling rice plants to survive flooding for weeks underwater and resume growth with conserved energy after water recession, as exemplified by the submergence-tolerant landrace “FR13A” (Figure 1B) [18,70,72,73]. In contrast, the escape strategy, or low-oxygen escape syndrome (LOES), involves the rapid elongation of leaves and internodes in order to grow above the water surface, thereby maintaining gas exchange and photosynthesis, as observed in deepwater rice cultivars such as “C9285” (Figure 1C) [17,74]. Group VII ETH response factors (ERFVIIs), including Submergence 1A-1 (Sub1A-1) and SNORKEL1/2 (SK1/2), are the central regulators of LOQS and LOES, respectively [72,73,74]. *Sub1A-1* promotes endurance of submergence by upregulating expression and activity of ADHs and pyruvate decarboxylases (PDCs) to sustain anaerobic energy metabolism, while simultaneously repressing the transcript accumulation of starch-degrading enzymes and sucrose synthases, thereby limiting carbohydrate consumption under submergence [67,70,75]. Although many lowland rice cultivars lacking *Sub1A-1* or *SK1*/*2* can exhibit internode elongation under submergence, most of them fail to reach the water surface (Figure 1A). As sustained elongation is highly energy-consuming, these plants often fail to recover upon de-submergence, suffering severe damage or death [11,17,67].

## 5. ROS Burst and Antioxidant Systems Triggered by Flooding Stress

Anaerobic respiration not only generates far less ATP than aerobic respiration but also leads to the accumulation of toxic metabolites such as intermediate acetaldehyde, ethanol, and lactate, thereby resulting in cytoplasmic acidification and further cellular damage. This energy crisis not only suppresses normal growth and development, but also triggers a cascade of secondary stresses, among which the burst of ROS represents a critical component.

ROS are unavoidable by-products of cellular metabolism and, at well-controlled levels, function as integral signaling molecules in the regulation of various biological processes such as plant growth, development, and stress responses [76,77]. However, under stress conditions, the excessive accumulation of ROS can cause toxic oxidative damage to DNA, RNA, proteins, and lipids [77,78,79]. During water stress, the sharp decline in oxygen availability can disrupt the mitochondrial electron transport chain, leading to electron leakage and ROS generation. Under hypoxic conditions, ROS overproduction also probably occurs in the chloroplasts and peroxisomes. Meanwhile, plasma membrane-bound NADPH oxidases, also known as RBOHs, are activated and utilize cytosolic NADPH as an electron donor to catalyze the production of superoxide anions in the apoplast. These superoxide radicals can be subsequently converted into hydrogen peroxide (H_2_O_2_) by superoxide dismutase (SOD) [17,76,77,80,81,82].

Upon flood recession, oxygen is rapidly reintroduced, and plant tissues that have adapted to prolonged O_2_ deficiency abruptly transition from an anoxic to an aerobic state. This process can be particularly destructive, causing reoxygenation injury, photoinhibition, and accelerated senescence, etc. During hypoxia, the large amounts of reducing equivalents such as NADH accumulate within cells. Upon reoxygenation and reillumination, excessive electron leakage from the mitochondrial electron transport chain, proton leakage in mitochondrial matrixes, reactivated photosynthetic metabolism in the chloroplasts, and increased activity of RBOHD result in a burst of ROS production. Additional ROS production results from the excessive oxygen reacting with ROS-associated by-products generated during flooding. Excessive ROS overwhelms the plant’s antioxidant capacity of ROS-scavenging mechanisms, including enzymatic and nonenzymatic antioxidants (i.e., SOD, catalase, ascorbate and glutathione), ultimately causing extensive cellular damage and tissue necrosis [9,17,18,79,81,83,84]. Therefore, post-submergence survival depends not only on tolerance to hypoxia but also largely on the capacity to cope with oxidative stress.

Consequently, submergence stress should not be viewed as a single event, but rather as a trigger for a complex stress network, including oxidative stress. To survive flooding, rice must effectively manage the “double-edged sword” nature of ROS. ROS serve as signaling molecules that activate adaptive responses, such as the induction of aerenchyma and AR formation. However, ROS over-accumulation must also be tightly controlled to prevent oxidative damage. This requires the precise coordination and fine regulation of ROS production and scavenging systems to maintain cellular redox homeostasis [77,81,85].

## 6. Regulatory Roles of Phytohormones

**Figure 3:**
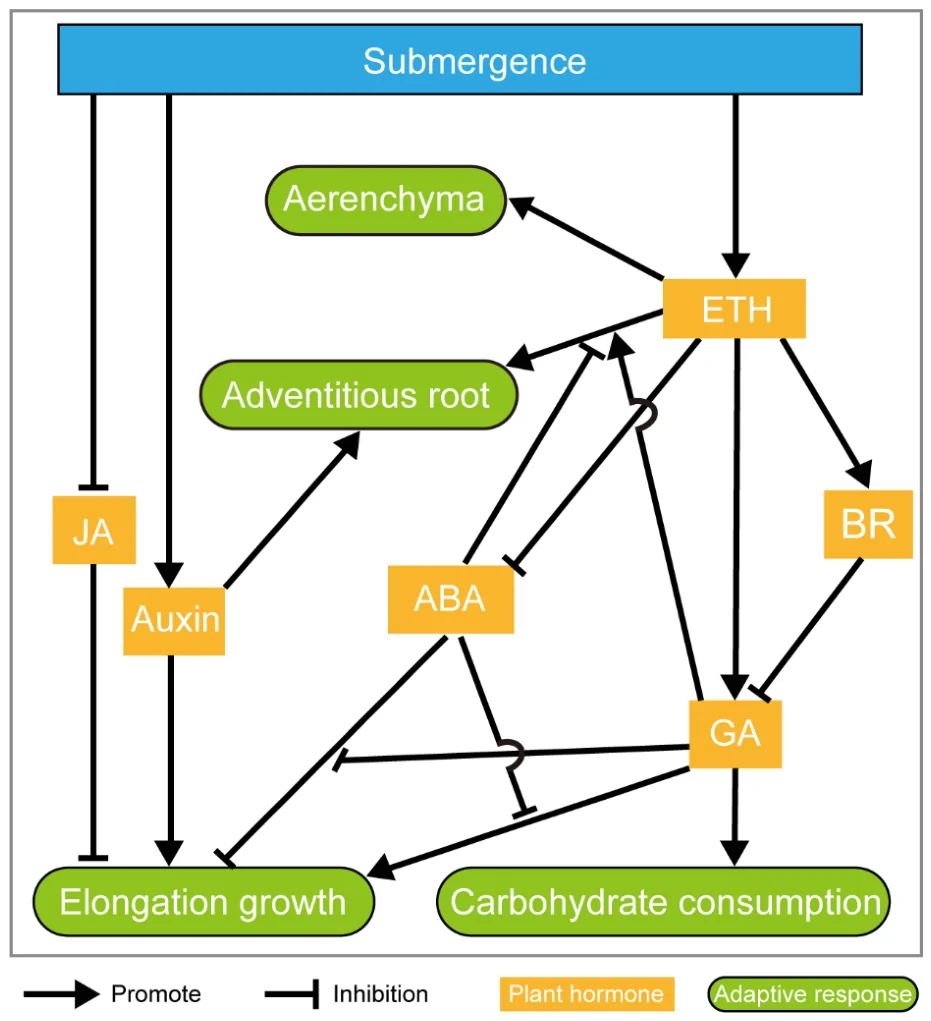
Interactions and crosstalk of different plant hormones in the adaptive response of rice to submergence. Pointed arrows represent positive regulation or promotion, whereas flat bar-ended lines indicate negative regulation or suppression.

Phytohormones play an indispensable role in the rice adaptive responses to submergence stress by regulating morphological, physiological, and molecular processes, thereby enabling plants to perceive, respond to, and acclimate to flooding environments (Figure 3) [79].

ETH is the only gaseous phytohormone, characterized by poor dissolution and a restricted diffusion rate in water, which contributes to its quick and invariable accumulation in submerged plant tissues [74,86]. In addition, submergence stress induces the transcription of genes encoding ACS and ACO, thereby promoting the conversion of the precursor S-adenosylmethionine (SAM) into ACC and subsequently into ETH [17,29,87,88]. Elevated endogenous ETH levels directly regulate structural adaptations such as aerenchyma formation, adventitious root development, and internode elongation, collectively contributing to rice endurance of submergence [5,29,49,55,74,86].

Other phytohormones, including ABA, GA, auxin, brassinosteroid (BR), and jasmonic acid (JA), also play critical roles under submergence stress [23,49,89,90,91,92]. The ETH-promoted adventitious root growth is enhanced by GA. Still, the effect is reduced by ABA, and an increased GA/ABA ratio also serves as a direct driving force for internode elongation [49,51,93]. Auxin is involved in ethylene-mediated adventitious root development by auxin signaling and PIN-FORMED (PIN)-mediated polar transport [49]. Also, auxin plays a positive role in regulating coleoptile length in rice under submergence. Moreover, submergence elevated the level of endogenous indole-3-acetic acid (IAA) in mature leaves, while the application of auxin transport inhibitors restricted coleoptile and seedling elongation [23,91,92]. Additionally, increased ethylene concentration during submergence enhanced BR levels and BR signaling by upregulating the expression of BR biosynthetic (*DWF1* and *DWF4*) and BR-signaling (*BZR3*) genes in a *Sub1A-1*-dependent manner [90]. Endogenous JA content reduced due to the increased expression level of a cytochrome P450 enzyme gene during submergence, which may inactivate jasmonate-isoleucine (JA-Ile). At the same time, exogenous JA was found to suppress submergence-induced internode elongation, suggesting that JA also functions in rice’s response to submergence [91,94,95].

Complex crosstalk among phytohormones, including ETH, auxin, GA, ABA, BR, and JA, coordinately regulates the overall adaptive response of rice to submergence (Figure 3). GA counteracts the growth inhibition of submerged rice internodes caused by ABA [93,96,97]. Under submerged conditions, the rapid decline in ABA levels in rice shoots is partly governed by ETH-induced expression of *OsABA8ox1*, a gene that encodes ABA 8′-hydroxylase, thereby promoting internode elongation [97,98]. In the escape strategy, ETH acts synergistically with GA to drive rapid stem elongation by promoting the accumulation or signaling of GA, enabling plants to reach the water surface and maintain the gas exchange [5,70]. In contrast, in the quiescence strategy, ETH induces the expression of *Sub1A-1*, which suppresses GA responsiveness and temporarily stunts growth, allowing plants to restrict energy consumption and survive submergence [5,23,70,96]. BR antagonizes the effects of GA by promoting the expression of the GA catabolic gene (*GA2ox7*), thereby reducing GA levels. Moreover, BR induces and/or stabilizes Slender Rice-1 (SLR1), which negatively regulates GA response [90]. IAA-JA balance and homeostasis, which are regulated by the RR26-*DS1* module through the shikimate pathway, play a vital role in post-germination growth in rice under submergence conditions. Elevated IAA levels and reduced JA accumulation facilitate robust seedling emergence and growth, significantly enhancing submergence tolerance [99].

## 7. Gene Regulatory Networks Underlying Rice Responses to Submergence

Hypoxic conditions induced by submergence trigger a series of physiological and metabolic responses in rice, including: (1) the formation of specialized tissues, such as aerenchyma, to enhance internal oxygen acquisition; (2) energy production through glycolysis and anaerobic fermentation; (3) growth suppression and energy conservation followed by recovery after flood recession, known as LOQS; and (4) the rapid internode elongation mediated by the ETH-GA signaling pathway, enabling shoots to escape submergence and restore gas exchange, referred to as LOES [100,101,102,103,104,105]. These adaptive responses are essentially orchestrated by the intricate and highly coordinated gene regulatory networks. In recent years, a suite of key submergence-tolerance genes has been identified through quantitative trait locus (QTL) mapping, association analysis, and map-based cloning approaches (Table 1).

### 7.1. Sub1A-Mediated Quiescence Strategy

*Sub1A*, originally identified from the *indica* cultivar “FR13A”, encodes an ERFVII transcription factor and is currently the most widely deployed submergence-tolerance gene in rice breeding programs [23,67,73,103]. Under submergence conditions, rapid ETH accumulation activates the expression of its tolerant allele, *Sub1A-1* [23,67,73]. Subsequently, *Sub1A-1* constrains further ETH production via a negative feedback loop and dampens transcript accumulation of senescence-associated genes, thereby delaying chlorophyll degradation and senescence [5,23,106,129,130]. Concurrently, *Sub1A-1* suppresses ETH-promoted GA responsiveness by increasing the accumulation of GA signaling repressors such as Slender Rice 1 (SLR1) and SLR1 Like 1 (SLRL1), effectively restraining shoot elongation and carbohydrate consumption [96].

However, unlike other ERFVIIs, Sub1A-1 resists degradation via oxygen-sensitive N-end rule proteolysis, allowing it to remain stable under both normoxic and hypoxic conditions and to function in multiple abiotic stress responses beyond submergence [108]. To specifically trigger hypoxia-related genes during flooding, Sub1A-1 transcriptionally activates two downstream ERFVII genes: *ERF66* and *ERF67*. ERF66 and ERF67 contain the conserved N-degron sequence and are substrates of the N-end rule pathway. They are degraded under normoxic abiotic stress conditions, but are stabilized and accumulated only under hypoxia. The regulatory cascade consisting of *Sub1A-1*, *ERF66*, and *ERF67* modulates a group of hypoxia response genes, including anaerobic metabolism genes, to enhance underwater tolerance [23,108].

Sub1A-1 further positively regulates inundation tolerance by directly binding to the promoter of *Mitogen-Activated Protein Kinase 3* (*OsMPK3*), thereby promoting its transcription. Overexpression of *OsMPK3* in Swarna-Sub1 elevates the expression of *SLR1* and *SLRL1*, reinforcing GA suppression. Notably, the variable region C-terminal to the ERF domain in tolerant Sub1A-1 contains a putative MAPK phosphorylation site, which can be phosphorylated and activated by OsMPK3. Activated Sub1A-1 further induces *OsMPK3* expression, forming a positive feedback loop that synergistically suppresses GA-mediated growth and enhances submergence tolerance [107].

In addition, *Sub1A-1* induces the expression of BR biosynthesis genes *DWARF1* (*DWF1*) and *DWF4*, as well as the BR signaling gene *BRASSINAZOLE RESISTANT 3* (*BZR3*). This leads to increased BR levels and enhanced BR sensitivity in signaling and perception [90]. Increased BR, in turn, upregulates *Sub1A-1* expression and induces the expression of the GA catabolic enzyme gene *GA2-oxidase 7* (*GA2ox7*), while also inducing and/or stabilizing SLR1 in the *Sub1A-1* plants. This further reduces bioactive GA accumulation and represses GA signaling to limit shoot elongation [90]. *Sub1A-1* also enhances the expression of antioxidant enzyme-encoding genes, thereby limiting ROS accumulation in aerial tissues and alleviating oxidative injury during de-submergence reoxygenation [9,131]. Introgression of *Sub1A-1* into elite cultivars confers strong submergence tolerance for up to two weeks without yield penalties [73,132].

### 7.2. Escape Strategy in Deepwater Rice

In contrast to the quiescence strategy mediated by *Sub1A-1*, deepwater rice adopts an escape strategy characterized by rapid internode elongation that allows leaves and shoots to emerge above the water surface, thereby restoring oxygen uptake during prolonged submergence (Figure 1C) [5,17,22,70]. Two ETH-responsive genes, *SK1* and *SK2*, cloned from the deepwater rice cultivar “C9285”, encode ERFVIIs and serve as the master regulators of internode elongation in the escape strategy. *SK1* and *SK2* show expression in the leaf blade and leaf sheath, as well as basal regions of the stem including nodes and internodes, and are greatly upregulated under deepwater conditions and ETH treatment. Additionally, the ETH signaling regulator *ETHYLENE INSENSITIVE 3* (*EIN3*)*-LIKE 1b* (*OsEIL1b*) can bind to the EIN3-binding sites of the *SK1* and *SK2* promoters, and may activate their expression as a transcription factor, thereby driving the rapid elongation in internodes [74,109,133]. Consequently, plant height increases dramatically within a short period, enabling escape from hypoxic conditions and the resumption of normal photosynthesis and respiration [74,109,111,134].

Although both deepwater and non-deepwater rice varieties accumulate ETH under submergence conditions, cultivars such as Taichung 65 (T65) and Nipponbare (NIP) lack functional *SK1* and *SK2* and have lost the ability to upregulate *SNORKEL-LIKE 1* (*SKL1*) under hypoxia, rendering them incapable of effective internode elongation [74,109,135].

### 7.3. Regulators of Early Internode Elongation

Deepwater rice also possesses the unique ability to elongate its internodes during the early stages of vegetative growth without transition from vegetative to reproductive growth (Figure 2D) [109,111,136]. This capacity is enhanced by submergence, ETH, and GA [74,109,111,134]. Comparative analyses revealed a 1 bp insertion/deletion between deepwater rice C9285 and normal paddy rice T65 in the *ACCELERATOR OF INTERNODE ELONGATION 1* (*ACE1*) gene. Submergence stress and GA treatment can induce the expression of *ACE1^C9285^* in deepwater rice plants, while *ACE1^T65^* expression remains relatively low under GA treatment or deepwater conditions. Overexpression of *ACE1^C9285^* activates cell division-related genes like *HISTONE H4* and *CDKA1* to confer cells of the intercalary meristem with the competence for cell division, thereby promoting internode elongation in the presence of GA [111].

Conversely, *DECELERATOR OF INTERNODE ELONGATION 1* (*DEC1*), also known as *PREMATURE INTERNODE ELONGATION 1* (*PINE1*), acts as a negative regulator of internode elongation. Its expression declines during the transition from vegetative to reproductive growth, thereby facilitating internode elongation in the T65 variety [111,112]. Although sequence polymorphisms, including insertions, deletions, and substitutions, exist between *DEC1* alleles in C9285 and T65, both alleles are functional. GA treatment or deepwater conditions reduce *DEC1* expression only in C9285, suggesting that differential expression regulation of *DEC1* contributes to cultivar-specific elongation responses [111].

The “Green Revolution” gene, *SEMIDWARF1* (*SD1*), encodes GA 20-oxidase 2 (OsGA20ox2), a key enzyme in GA biosynthesis. Multiple *sd1* alleles with defective OsGA20ox2 activities reduce bioactive GA production, resulting in semi-dwarfism, enhanced lodging resistance, and increased yield [137,138,139]. *SD1^C9285^* is a submergence-tolerance gene cloned from deepwater rice variety C9285. Under submerged conditions, deepwater rice carrying *SD1^C9285^* exhibits strongly elevated *SD1* expression and higher enzymatic activity of OsGA20ox2 than normal paddy rice variety T65 with *SD1^T65^*, leading to higher amounts of active GA accumulation and extreme internode elongation. Moreover, the ETH signaling transcription factor EIN3-LIKE 1a (OsEIL1a) directly transcriptionally activated *SD1^C9285^*, which acts synergistically with *SK1*/*2* to promote rapid shoot elongation [110].

*OsACS5*, encoding an ACS, is upregulated by GA and downregulated by ABA under hypoxia, and promotes the sustained elongation growth of deepwater rice during submergence by regulating ETH biosynthesis [87]. *OsUsp1* (*universal stress protein 1*), a gene-encoding universal stress protein identified from deepwater rice, is strongly induced in the youngest growth-responsive internode following submergence and ETH treatment [113]. *Oryza sativa hypoxia induced Gene Domain 2* (*OsHIGD2*), encoding a hypoxia-responsive mitochondrial protein, is involved in an ETH-independent internode elongation pathway and is rapidly induced during the early onset of hypoxia response before ETH accumulation. By directly responding to oxygen deficiency, *OsHIGD2* may bridge the gap between stress perception and hormonal regulation within the submergence-tolerance network [114].

### 7.4. Regulators of Anaerobic Germination (AG) and Anaerobic Seedling Development (ASD)

Flooding can cause poor germination, impaired seedling establishment, and weak seedling growth due to hypoxia or anoxia of rice seeds [4,140]. AG tolerance enabling uniform and robust germination and seedling establishment is characterized by the rapid elongation of the coleoptile and the concurrently dampened growth of the radicle under submerged conditions (Figure 2A) [116,120]. Hypoxia and sugar starvation signals induced by flooding activate *calcineurin B-like* (*CBL*)*-interacting protein kinase 15* (*OsCIPK15*), which sequentially activates sucrose nonfermenting 1 (Snf1)-related protein kinase 1A (SnRK1A) and the transcription factor myeloblastosis sugar response complex 1 (MYBS1). This phosphorylation cascade induces the expression of *RAmy3D*, encoding α-amylase, thereby mobilizing stored starch into fermentable sugars to fuel anaerobic respiration for AG and ASD [66,115,117,118,141]. Rice calcineurin B-like protein10 (OsCBL10) mediates AG tolerance by regulating the calcium flux, OsCIPK15 protein abundance, and its downstream cascade during seed germination and coleoptile elongation [119]. *OsTPP7*, encoding trehalose-6-phosphate (T6P) phosphatase, modulates T6P turnover and local sucrose availability to promote α-amylase activity, thereby enhancing AG tolerance [116,120]. As a heterotrimeric G protein γ3 subunit, RGG3/GS3 promotes anaerobic coleoptile elongation during rice seed germination. Mechanistically, *RGG3*/*GS3* induces the expression of T6P phosphatases (*TPP1*/*TPP7*) and downregulates T6P levels, and also positively regulates CIPK15–SnRK1A–MYBS1 and hormone-responsive signaling pathways to govern α-amylase gene expression [121]. A feed-forward regulatory loop involving the mitochondria-localized alternative oxidase (AOX1a) and H_2_O_2_ has been shown to promote hypoxic germination and seedling growth/development under flooded conditions via the CIPK15-SnRK1A-MYBS1 cascade [122].

OsGF14h, a 14-3-3 protein identified from weedy rice, functions as a signal switch to suppress ABA signaling and activate GA biosynthesis through interacting with a HD-Zip II family transcription factor OsHOX3 and VIVIPAROUS1 (OsVP1), thereby markedly promoting AG tolerance and ASD [123]. *Acireductone dioxygenase 1* (*OsARD1*) is highly induced by submergence and participates in the methionine salvage pathway to produce the substrate of ETH synthesis. Overexpression of *OsARD1* increases endogenous ETH production and promotes the elongation of the shoot and coleoptile, thereby enhancing the submergence tolerance [124]. OsUGT75A, a UDP-glucosyltransferase, reduces the levels of free ABA and JA through glycosylation and mediates the interactions between JASMONATE ZIMDOMAINs (OsJAZs) and ABSCISIC ACID-INSENSITIVEs (OsABIs), thereby enhancing coleoptile elongation under submerged conditions [125]. *qSHS5*, encoding a REX1 DNA excision repair protein, contributes to submergence tolerance in terms of seedling height and establishment under submerged conditions by facilitating the repair of oxidative DNA damage, maintaining cell proliferation, and regulating ROS homeostasis [126].

*miR393a* suppresses coleoptile growth by negatively regulating the auxin nuclear receptor genes *auxin transport inhibitor resistant 1* (*OsTIR1*) and *auxin signaling F-box 2* (*OsAFB2*). During seed germination and seedling establishment, the inhibition of *miR393a* induces the auxin response, thereby promoting coleoptile elongation under submergence. Exogenous ABA treatment promotes *miR393* expression and represses coleoptile elongation [127]. Through comparative transcriptional responses of rice submergence-tolerant and submergence-intolerant accessions, we have previously identified a key regulatory component, *OsEXPB3*. As a β-expansin family member, *OsEXPB3* was consistently upregulated in four tolerant varieties. Loss-of-function of *OsEXPB3* results in the repression of coleoptile and seedling elongation, as well as compromised submergence adaptation. Hormone determination assays revealed that *OsEXPB3* may be involved in coordinating the growth- and defense-related hormone balance to modulate submergence acclimation [128].

## 8. Submergence-Tolerant Rice Germplasms and Application of Submergence Tolerance Genes

The availability of flooding-tolerant rice germplasm resources not only provides a material basis for the in-depth analysis of the physiological and molecular mechanisms of rice response and adaptation to submergence stress, but also lays the foundation for the breeding and genetic improvement of submergence resilience in the future. To identify rice varieties that are tolerant or resilient to submergence and stagnant flooding, 6274 diverse genotypes of IRRI germplasm resources were effectively screened and eighty-nine outstanding flood-tolerant genotypes with broad genetic diversity were identified [142]. Based on the established evaluation criteria and multi-stage assessment system, we recently screened 189 rice accessions. We ultimately identified four germplasms with submergence tolerance—the *indica* “Minghui63” and “Diantun502”, and *japonica* “Funuo3” and “Huikenuo1701”—under various submergence scenarios across different stages: germination stage, seedling stage, and tillering stage [15].

Exploration, identification, and functional characterization of the superior genes involved in submergence tolerance using genetic, physiological, biochemical, molecular, and omics methods has provided key genetic resources and theoretical guidance for molecular breeding in rice. Through marker-assisted selection (MAS), the International Rice Research Institute (IRRI) and its partners have successfully introgressed the *Sub1A-1* gene into several widely cultivated mega varieties with submergence intolerance in South and Southeast Asia, such as Swarna, Samba Mahsuri, and IR64, leading to the development of submergence-tolerant cultivars including Swarna-Sub1, SambaMahsuri-Sub1, and IR64-Sub1 [4,23,73,132,143]. These improved varieties exhibit no significant differences in agronomic performance, yield, or grain quality compared with their recurrent parents under normal growth conditions. However, when exposed to short-term complete submergence, they show a pronounced survival advantage and are able to restore growth rapidly after the floodwaters subside. In addition, to meet the specific requirements of direct-seeded rice systems, the introgression or introduction of AG/ASD-tolerant genes, such as *OsTPP7* or weedy rice *OsGF14h*, into elite varieties has proven effective in mitigating flood-induced problems including poor and uneven seed germination and low seedling rate and vigor [120,123]. Introgression of *OsTPP7* into mega variety Ciherang-Sub1 harboring *Sub1A-1* resulted in improved AG tolerance from *OsTPP7* with the concomitant submergence tolerance from *Sub1A-1* [144]. The development of “smart” rice cultivars simultaneously carrying *SD1^H1^* and *qSHS5^H3^* could confer both submergence tolerance in terms of robust seedling establishment and high yield [126].

## 9. Conclusions and Perspectives

As an important staple crop, rice has evolved intricate and specialized structures to adapt to aquatic environments during long-term evolution and domestication, including LGF, aerenchyma, AR, and the ROL barrier. Some Asian rice varieties have also developed specific strategies to tolerate AG and ASD during germination and early seedling growth under flooded conditions. When subjected to destructive submergence stress, rice at the vegetative or adult stage has further developed two distinct survival strategies, quiescence and escape for stunted or elongated growth. The regulation of these acclimation strategies involves energy and carbohydrate metabolism, ROS homeostasis, the PCD process, biomacromolecule synthesis and deposition, hormone biosynthesis and signaling pathway, and other submergence-associated genes. However, many key players are still unidentified, and the relationship and interplay between these pathways remains largely elusive. Moreover, the identified rice germplasm resources for flooding-tolerance and favorable natural allelic genes, and corresponding molecular markers for submergence tolerance that can be directly used for genetic improvement, are still relatively scarce.

Further investigation is warranted to identify and characterize novel superior genes in genetically diverse wild rice and flooding-tolerant germplasms, which may adopt distinct mechanisms and strategies for submergence adaptation and tolerance. Also, new technologies such as single-cell sequencing, spatial transcriptomics, integrative multi-omics and artificial intelligence-assisted data mining should be utilized to comprehensively uncover the molecular regulatory hierarchy and landscape of rice mitigation and adaptation to flooding stress, providing gene resources and targets for rational design breeding to enhance submergence tolerance and resilience of rice and even other crops under increasingly complex and variable environments. With the rapid advancement of genome-editing technologies such as CRISPR/Cas9, fine-tuning key gene expression, knocking-in stress-responsive elements or creating superior allelic variants has become feasible, highlighting their potential as precise and smart molecular design breeding tools. Therefore, these identified flooding-tolerance genes can be used in genome editing to improve endurance of submergence and greatly accelerate the cultivation process of rice varieties resistant to submergence stress. Meanwhile, utilizing molecular breeding to pyramid AG/ASD-related favorable genes (i.e., *OsTPP7*, *OsGF14h*, *OsCBL10*, and *OsUGT75A*) and adult-stage tolerant allelic genes (i.e., *Sub1A-1* or *SK1*/*SK2*, *SD1^C9285^*, and *ACE1^C9285^*) may be an effective approach to enhance tolerance throughout rice’s entire growth period under flooded conditions. Ultimately, it is promising that future breeding strategies will focus on combining and balancing the submergence adaptability with desirable agronomic traits, such as improved plant architecture, efficient mineral utilization, and high nutrient content. This will facilitate the cultivation of new varieties with enhanced submergence tolerance, high and stable yield, superior quality, and resource-conserving and environmentally friendly characteristics.

## Figures and Tables

**Table 1:** Genes associated with submerged conditions in rice.

Gene	Description	Effect of Hypoxia Stress	Hormones Involved	Downstream Components	References
*LGF1*/*HSD1*	A wax synthesis gene	Formation of LGF and ROL barrier			[40,42,64]
*RBOH*	Respiratory burst oxidase homolog	ROS production and aerenchyma formation	ETH		[29,57,58]
*MT2b*	Metallothionein	ROS scavenging and formation of aerenchyma			[29,55]
*OsABA8ox1*	ABA 8′-hydroxylase	Submergence-induced ABA catabolism for stem elongation	ETH, ABA		[97,98]
*DS1*	The shikimate pathway entry enzyme DAHPS2	Promotes post-germination growth and rapid seedling emergence under submergence	IAA, JA		[99]
*Sub1A-1*	ERFVII gene	Submergence tolerance in quiescent strategy	ETH, GA, BR, JA, IAA	*ADH1*/*2*, *PDC2*/*4*, *Expansins*, *Sus1*/*2*/*3*, *RAmy3D*, *SLR1*, *SLRL1*, *ERF66*, *ERF67*, *DWF1*, *DWF4*, *OsBZR3*, *GA2ox7*	[23,38,67,73,90,96,106]
*OsSLR1*, *OsSLRL1*	GA signaling repressors	Suppress internode elongation and carbohydrate consumption	ETH, BR, GA		[90,96,107]
*OsERF66*, *OsERF67*	ERFVIIs	Only stabilized under hypoxia for submergence tolerance		Hypoxia-related genes	[108]
*MPK3*	Mitogen-activated protein kinase	*MPK3*-*Sub1A-1* forms a positive regulatory loop to enhance the submergence tolerance	ETH	*Sub1A-1*	[107]
*SK1*, *SK2*	ERFVIIs	Promote internode elongation in escape strategy	ETH, GA		[74,109]
*SD1^C9285^*	A GA synthesis enzyme OsGA20ox2	Promotes internode elongation by increasing active GA accumulation	ETH, GA		[109,110]
*ACE1^C9285^*	Accelerator of internode elongation	Accelerates the internode elongation through conferring cells of the intercalary meristem with the competence for cell division	GA	*HISTONE H4*, *CDKA1*	[111]
*DEC1*/*PINE1*	C2H2 Zinc finger TF, Decelerator of internode elongation	Suppresses internode elongation by regulating the activity of the intercalary meristem	GA	*HISTONE H4*, *CDKA1*	[111,112]
*OsEIL1a*	Ethylene-Insensitive 3-Like 1a	Relay of ETH signaling to GA biosynthesis by activating *SD1*	ETH, GA	*SD1^C9285^*	[110]
*OsEIL1b*	Ethylene-Insensitive 3-Like 1b	Escape strategy in deepwater rice by binding to the promoter regions of *SK1* and *SK2*	ETH	*SK1*, *SK2*	[74]
*OsACS5*	Ethylene biosynthesis	Promotes the sustained elongation growth of deepwater rice	ETH, ABA, GA		[87]
*OsUSP1*	MJ0577 subfamily of ATP-binding USP proteins	Induced by submergence and ETH	ETH		[113]
*OsHIGD2*	Hypoxia-responsive protein	Reduces hypoxia-driven respiratory damage in mitochondria by participating in the early stage of hypoxia signaling			[114]
*CIPK15*	Calcineurin B-like (CBL)-interacting protein kinase 15	CIPK15-SnRK1A-MYBS1 regulates carbohydrate catabolism and enables AG and ASD under flooding		*SnRK1A*, *MYBS1*, *RAmy3D*	[66,115,116,117,118]
*OsCBL10*	Calcineurin B-like protein 10	Affects AG tolerance by regulating OsCIPK15 protein abundance during flooding stress		*CIPK15*	[119]
*OsTPP7*	Trehalose-6-phosphate phosphatase	Regulates T6P turnover and local sucrose availability and enhances AG tolerance			[120]
*RGG3/GS3*	The heterotrimeric G protein γ3 subunit	Regulates CIPK15–SnRK1A–MYBS1 and hormone-responsive signaling pathways to promote AG		*TPP1/TPP7*, *CIPK15*–*SnRK1A*–*MYBS1*, *GAMYB*–*DOF3*	[121]
*AOX1a*	The mitochondria-localized alternative oxidase	Forms a feed-forward loop with H_2_O_2_ to promote hypoxic germination and seedling development		*CIPK15–SnRK1A–MYBS1*	[122]
*OsGF14h*	14-3-3 protein from weedy rice	Improves AG and ASD through balancing ABA signaling and GA biosynthesis	GA, ABA	*OsGA20ox1*	[123]
*OsARD1*	Acireductone dioxygenase	Promotes the elongation of the shoot and coleoptile owing to the increased ETH production	ETH	ACS genes	[124]
*OsUGT75A*	UDP-glucosyltransferase	Glycosylates ABA and JA to regulate the coleoptile length for AG tolerance	ABA, JA	*OsJAZs*, *OsABI3*/*5*	[125]
*qSHS5*	A nucleic acid excision repair protein	Regulates seedling height and seedling rate under submergence			[126]
miR393	Degradation of auxin receptors	Inhibits coleoptile growth by negatively regulating the auxin signaling pathway underwater	Auxin, ABA	*OsTIR1*, *OsAFB2*	[127]
*OsEXPB3*	β-expansin	Maintains the growth–defense balance during submergence	ETH, GA, ABA, JA, salicylic acid (SA)		[128]

## Data Availability

No new data were created or analyzed in this study. Data sharing is not applicable to this article.

## Abbreviations

The following abbreviations are used in this manuscript:

LGF	leaf gas film
AR	adventitious root
ROL	radial oxygen loss
ROS	reactive oxygen species
ETH	ethylene
GA	gibberellin
PCD	programmed cell death
H_2_O_2_	hydrogen peroxide
ABA	abscisic acid
ARF	auxin response factor
ACC	1-aminocyclopropane-1-carboxylic acid
ACS	1-aminocyclopropane-1-carboxylic acid synthase
ACO	ACC oxidase
RBOH	burst oxidase homolog
CDPKs	Ca^2+^-dependent protein kinases
SUS	sucrose synthase
LDH	lactate dehydrogenase
ADH	alcohol dehydrogenase
LOQS	low-oxygen quiescence syndrome
LOES	low-oxygen escape syndrome
ERF	ETH response factor
ERFVIIs	group VII ethylene response factors
PDC	pyruvate decarboxylase
SOD	superoxide dismutase
SAM	S-adenosylmethionine
JA	jasmonic acid
BR	brassinosteroid
SA	salicylic acid
AG	anaerobic germination
ASD	anaerobic seedling development
T6P	trehalose-6-phosphate
MAS	marker-assisted selection
IRRI	International Rice Research Institute

## References

[1] Garcia-Vila M., dos Santos Vianna M., Harrison M.T., Liu K., de S. Nóia-Júnior R., Weber T.K.D., Zhao J., Acutis M., Archontoulis S., Asseng S. (2025). Gaps and strategies for accurate simulation of waterlogging impacts on crop productivity. Nat. Food.

[2] Kim W., Iizumi T., Hosokawa N., Tanoue M., Hirabayashi Y. (2023). Flood impacts on global crop production: Advances and limitations. Environ. Res. Lett..

[3] Zhu G., Wu H., Chen Y., Mondal S., Ismail A.M. (2023). Growth characteristics and yield of contrasting rice genotypes under long-term stagnant flooding. Field Crops Res..

[4] Oumarou Mahamane A.R., Kamara J.S., Byiringiro M.H., Sow A. (2026). Exploring flooding challenges, causes, and mitigation strategies in rice. Int. J. Genom..

[5] Li G., Wei N., Hou H. (2026). Uncovering the secrets of how plants adapt to water stress. Plant Cell Environ..

[6] Wang J., Han M., Huang Y., Zhao J., Liu C., Ma Y. (2024). Flooding tolerance of rice: Regulatory pathways and adaptive mechanisms. Plants.

[7] Phukan U.J., Mishra S., Shukla R.K. (2015). Waterlogging and submergence stress: Affects and acclimation. Crit. Rev. Biotechnol..

[8] Miro B., Ismail A.M. (2013). Tolerance of anaerobic conditions caused by flooding during germination and early growth in rice (*Oryza sativa* L.). Front. Plant Sci..

[9] Yuan L.B., Chen M.X., Wang L.N., Sasidharan R., Voesenek L.A.C.J., Xiao S. (2023). Multi-stress resilience in plants recovering from submergence. Plant Biotechnol. J..

[10] Luo F.L., Thiele B., Janzik I., Zeng B., Schurr U., Matsubara S. (2012). De-submergence responses of antioxidative defense systems in two wetland plants having escape and quiescence strategies. J. Plant Physiol..

[11] Jackson M.B., Ram P.C. (2003). Physiological and molecular basis of susceptibility and tolerance of rice plants to complete submergence. Ann. Bot..

[12] Renziehausen T., Frings S., Schmidt-Schippers R. (2024). ‘Against all floods’: Plant adaptation to flooding stress and combined abiotic stresses. Plant J..

[13] Meng Y., Yu S.G., Yu Y.M., Jiang L.X. (2021). Flooding depth and duration concomitantly influence the growth traits and yield of rice. Irrig. Drain..

[14] Zhou W., Chen F., Meng Y., Chandrasekaran U., Luo X., Yang W., Shu K. (2020). Plant waterlogging/flooding stress responses: From seed germination to maturation. Plant Physiol. Biochem..

[15] Liu S.Q., Ai H., Duan S.M., Li R.N., Owusu E.A., Lv L.L., Sun Z.L., Xu D.C., Zhang M.Z., Zhou A.F. (2026). Integrated analysis of laboratory and field experiments to screen rice germplasm for submergence tolerance across various growth stages. Field Crops Res..

[16] Angaji S.A., Septiningsih E.M., Mackill D.J., Ismail A.M. (2009). QTLs associated with tolerance of flooding during germination in rice (*Oryza sativa* L.). Euphytica.

[17] Bailey-Serres J., Voesenek L.A. (2008). Flooding stress: Acclimations and genetic diversity. Annu. Rev. Plant Biol..

[18] Nakamura M., Noguchi K. (2020). Tolerant mechanisms to O_2_ deficiency under submergence conditions in plants. J. Plant Res..

[19] Kato Y., Collard B.C., Septiningsih E.M., Ismail A.M. (2014). Physiological analyses of traits associated with tolerance of long-term partial submergence in rice. AoB Plants.

[20] Fu J., Jian Y., Wang X., Li L., Ciais P., Zscheischler J., Wang Y., Tang Y., Müller C., Webber H. (2023). Extreme rainfall reduces one-twelfth of China’s rice yield over the last two decades. Nat. Food.

[21] Vergara G.V., Nugraha Y., Esguerra M.Q., Mackill D.J., Ismail A.M. (2014). Variation in tolerance of rice to long-term stagnant flooding that submerges most of the shoot will aid in breeding tolerant cultivars. AoB Plants.

[22] Phukan U.J., Jindal S., Laldinsangi C., Singh P.K., Longchar B. (2023). A microscopic scenario on recovery mechanisms under waterlogging and submergence stress in rice. Planta.

[23] Khalil M.I., Hassan M.M., Samanta S.C., Chowdhury A.K., Hassan M.Z., Ahmed N.U., Somaddar U., Ghosal S., Robin A.H.K., Nath U.K. (2024). Unraveling the genetic enigma of rice submergence tolerance: Shedding light on the role of ethylene response factor-encoding gene *SUB1A*-*1*. Plant Physiol. Biochem..

[24] Tamang B.G., Fukao T. (2015). Plant adaptation to multiple stresses during submergence and following desubmergence. Int. J. Mol. Sci..

[25] Sone C., Sakagami J.I. (2017). Physiological mechanism of chlorophyll breakdown for leaves under complete submergence in rice. Crop Sci..

[26] Chen L.Q., Cheung L.S., Feng L., Tanner W., Frommer W.B. (2015). Transport of Sugars. Annu. Rev. Biochem..

[27] Lee H.S., Hwang W.H., Jeong J.H., Ahn S.H., Baek J.S., Jeong H.Y., Park H.K., Ku B.I., Yun J.T., Lee G.H. (2019). Analysis of the distribution of assimilation products and the characteristics of transcriptomes in rice by submergence during the ripening stage. BMC Genom..

[28] Tong C., Hill C.B., Zhou G., Zhang X.Q., Jia Y., Li C. (2021). Opportunities for improving waterlogging tolerance in cereal crops—Physiological traits and genetic mechanisms. Plants.

[29] Yamauchi T., Colmer T.D., Pedersen O., Nakazono M. (2018). Regulation of root traits for internal aeration and tolerance to soil waterlogging-flooding stress. Plant Physiol..

[30] Ismail A.M. (2018). Submergence tolerance in rice: Resolving a pervasive quandary. New Phytol..

[31] Herzog M., Striker G.G., Colmer T.D., Pedersen O. (2016). Mechanisms of waterlogging tolerance in wheat-a review of root and shoot physiology. Plant Cell Environ..

[32] Zhang R., Xuan L., Ni L., Yang Y., Zhang Y., Wang Z., Yin Y., Hua J. (2023). *ADH* gene cloning and identification of flooding-responsive genes in *Taxodium distichum* (L.) Rich. Plants.

[33] Gao Y., Sui D., Shi S., Zou J., Wang S., Sun L., Lei C., Li S., Wang H. (2025). Physiological and molecular adaptation of the ahuehuete (*Taxodium mucronatum* Ten.) to waterlogging. Plants.

[34] Liu Z., Zhang L., Huangfu Y., Chen W., Xie Z., Tian B., Wu T., Cao G., Guo J., Wei F. (2025). The synergistic response of *Arundo donax* to multiple stressors: New insights from root genome-wide transcription analysis. Ind. Crops Prod..

[35] Jackson M.B., Colmer T.D. (2005). Response and adaptation by plants to flooding stress. Ann. Bot..

[36] Pedersen O., Rich S.M., Colmer T.D. (2009). Surviving floods: Leaf gas films improve O_2_ and CO_2_ exchange, root aeration, and growth of completely submerged rice. Plant J..

[37] Colmer T.D., Pedersen O. (2008). Underwater photosynthesis and respiration in leaves of submerged wetland plants: Gas films improve CO_2_ and O_2_ exchange. New Phytol..

[38] Chakraborty K., Guru A., Jena P., Ray S., Guhey A., Chattopadhyay K., Sarkar R.K. (2021). Rice with *SUB1* QTL possesses greater initial leaf gas film thickness leading to delayed perception of submergence stress. Ann. Bot..

[39] Winkel A., Pedersen O., Ella E., Ismail A.M., Colmer T.D. (2014). Gas film retention and underwater photosynthesis during field submergence of four contrasting rice genotypes. J. Exp. Bot..

[40] Kurokawa Y., Nagai K., Huan P.D., Shimazaki K., Qu H., Mori Y., Toda Y., Kuroha T., Hayashi N., Aiga S. (2018). Rice leaf hydrophobicity and gas films are conferred by a wax synthesis gene (*LGF1*) and contribute to flood tolerance. New Phytol..

[41] Samuels L., Kunst L., Jetter R. (2008). Sealing plant surfaces: Cuticular wax formation by epidermal cells. Annu. Rev. Plant Biol..

[42] Zhang Z., Cheng Z.J., Gan L., Zhang H., Wu F.Q., Lin Q.B., Wang J.L., Wang J., Guo X.P., Zhang X. (2016). *OsHSD1*, a hydroxysteroid dehydrogenase, is involved in cuticle formation and lipid homeostasis in rice. Plant Sci..

[43] Sauter M. (2013). Root responses to flooding. Curr. Opin. Plant Biol..

[44] Karlova R., Boer D., Hayes S., Testerink C. (2021). Root plasticity under abiotic stress. Plant Physiol..

[45] Lin C., Zhu T., Ogorek L.L.P., Wang Y., Sauter M., Pedersen O. (2022). The pyramiding of three key root traits aid breeding of flood-tolerant rice. Plants.

[46] Colmer T.D. (2003). Long-distance transport of gases in plants: A perspective on internal aeration and radial oxygen loss from roots. Plant Cell Environ..

[47] Meng F., Xiang D., Zhu J., Li Y., Mao C. (2019). Molecular mechanisms of root development in rice. Rice.

[48] Lorbiecke R., Sauter M. (1999). Adventitious root growth and cell-cycle induction in deepwater rice. Plant Physiol..

[49] Steffens B., Rasmussen A. (2016). The physiology of adventitious roots. Plant Physiol..

[50] Steffens B., Kovalev A., Gorb S.N., Sauter M. (2012). Emerging roots alter epidermal cell fate through mechanical and reactive oxygen species signaling. Plant Cell.

[51] Steffens B., Wang J., Sauter M. (2005). Interactions between ethylene, gibberellin and abscisic acid regulate emergence and growth rate of adventitious roots in deepwater rice. Planta.

[52] Steffens B., Sauter M. (2009). Epidermal cell death in rice is confined to cells with a distinct molecular identity and is mediated by ethylene and H_2_O_2_ through an autoamplified signal pathway. Plant Cell.

[53] Armstrong W. (1979). Aeration in higher plants. Adv. Bot. Res..

[54] Drew M.C., He C.J., Morgan P.W. (2000). Programmed cell death and aerenchyma formation in roots. Trends Plant Sci..

[55] Steffens B., Geske T., Sauter M. (2011). Aerenchyma formation in the rice stem and its promotion by H_2_O_2_. New Phytol..

[56] Yamauchi T., Tanaka A., Inahashi H., Nishizawa N.K., Tsutsumi N., Inukai Y., Nakazono M. (2019). Fine control of aerenchyma and lateral root development through AUX/IAA- and ARF-dependent auxin signaling. Proc. Natl. Acad. Sci. USA.

[57] Yamauchi T., Nakazono M. (2022). Mechanisms of lysigenous aerenchyma formation under abiotic stress. Trends Plant Sci..

[58] Yamauchi T., Yoshioka M., Fukazawa A., Mori H., Nishizawa N.K., Tsutsumi N., Yoshioka H., Nakazono M. (2017). An NADPH oxidase RBOH functions in rice roots during lysigenous aerenchyma formation under oxygen-deficient conditions. Plant Cell.

[59] Yamauchi T., Fukazawa A., Nakazono M. (2017). *METALLOTHIONEIN* genes encoding ROS scavenging enzymes are down-regulated in the root cortex during inducible aerenchyma formation in rice. Plant Signal. Behav..

[60] Colmer T.D. (2003). Aerenchyma and an inducible barrier to radial oxygen loss facilitate root aeration in upland, paddy and deep-water rice (*Oryza sativa* L.). Ann. Bot..

[61] Nishiuchi S., Yamauchi T., Takahashi H., Kotula L., Nakazono M. (2012). Mechanisms for coping with submergence and waterlogging in rice. Rice.

[62] Peralta Ogorek L.L., Jiménez J.C., Visser E.J.W., Takahashi H., Nakazono M., Shabala S., Pedersen O. (2024). Outer apoplastic barriers in roots: Prospects for abiotic stress tolerance. Funct. Plant Biol..

[63] Shiono K., Yamauchi T., Yamazaki S., Mohanty B., Malik A.I., Nagamura Y., Nishizawa N.K., Tsutsumi N., Colmer T.D., Nakazono M. (2014). Microarray analysis of laser-microdissected tissues indicates the biosynthesis of suberin in the outer part of roots during formation of a barrier to radial oxygen loss in rice (*Oryza sativa*). J. Exp. Bot..

[64] Jiménez J.C., Noorrohmah S., Suresh K., Zeisler-Diehl V.V., Peralta Ogorek L.L., Herzog M., Pellegrini E., Nagai K., Ashikari M., Takahashi H. (2024). *Leaf Gas Film 1* promotes glycerol ester accumulation and formation of a tight root barrier to radial O_2_ loss in rice. Plant Physiol..

[65] Geigenberger P. (2003). Response of plant metabolism to too little oxygen. Curr. Opin. Plant Biol..

[66] Adak M.K., Das A., Kundu A., Chatterjee M., Hasanuzzaman M. (2023). Molecular mechanisms in understanding anoxia tolerance in rice seeds under submergence and their implication in rice biotechnology. Seeds.

[67] Fukao T., Xu K., Ronald P.C., Bailey-Serres J. (2006). A variable cluster of ethylene response factor-like genes regulates metabolic and developmental acclimation responses to submergence in rice. Plant Cell.

[68] Shingaki-Wells R., Millar A.H., Whelan J., Narsai R. (2014). What happens to plant mitochondria under low oxygen? An omics review of the responses to low oxygen and reoxygenation. Plant Cell Environ..

[69] Lim M.N., Lee S.E., Jeon J.S., Yoon I.S., Hwang Y.S. (2024). OsbZIP38/87-mediated activation of *OsHXK7* improves the viability of rice cells under hypoxic conditions. J. Plant Physiol..

[70] Nagai K., Hattori Y., Ashikari M. (2010). Stunt or elongate? Two opposite strategies by which rice adapts to floods. J. Plant Res..

[71] Voesenek L.A., Bailey-Serres J. (2013). Flooding tolerance: O_2_ sensing and survival strategies. Curr. Opin. Plant Biol..

[72] Voesenek L.A.C.J., Bailey-Serres J. (2015). Flood adaptive traits and processes: An overview. New Phytol..

[73] Xu K., Xu X., Fukao T., Canlas P., Maghirang-Rodriguez R., Heuer S., Ismail A.M., Bailey-Serres J., Ronald P.C., Mackill D.J. (2006). *Sub1A* is an ethylene-response-factor-like gene that confers submergence tolerance to rice. Nature.

[74] Hattori Y., Nagai K., Furukawa S., Song X.J., Kawano R., Sakakibara H., Wu J., Matsumoto T., Yoshimura A., Kitano H. (2009). The ethylene response factors *SNORKEL1* and *SNORKEL2* allow rice to adapt to deep water. Nature.

[75] John D., Shylaraj K.S. (2020). Mechanism of anoxic tolerance in backcross lines developed through Jyothi x Swarna-Sub 1 under submergence stress. Indian J. Genet..

[76] Baxter A., Mittler R., Suzuki N. (2014). ROS as key players in plant stress signalling. J. Exp. Bot..

[77] Mittler R. (2017). ROS are good. Trends Plant Sci..

[78] Mittler R. (2002). Oxidative stress, antioxidants and stress tolerance. Trends Plant Sci..

[79] Wang L.N., Wang W.C., Liao K., Xu L.J., Xie D.X., Xie R.H., Xiao S. (2025). Survival mechanisms of plants under hypoxic stress: Physiological acclimation and molecular regulation. J. Integr. Plant Biol..

[80] Qi X., Li Q., Ma X., Qian C., Wang H., Ren N., Shen C., Huang S., Xu X., Xu Q. (2019). Waterlogging-induced adventitious root formation in cucumber is regulated by ethylene and auxin through reactive oxygen species signalling. Plant Cell Environ..

[81] Sasidharan R., Hartman S., Liu Z., Martopawiro S., Sajeev N., van Veen H., Yeung E., Voesenek L.A.C.J. (2018). Signal dynamics and interactions during flooding stress. Plant Physiol..

[82] Renziehausen T., Chaudhury R., Hartman S., Mustroph A., Schmidt-Schippers R.R. (2024). A mechanistic integration of hypoxia signaling with energy, redox, and hormonal cues. Plant Physiol..

[83] Geng R., Xu M., Xu L., Yan G., Cai G. (2026). Biological mechanisms of waterlogging tolerance in plants. Plant Cell Environ..

[84] Yeung E., Bailey-Serres J., Sasidharan R. (2019). After the deluge: Plant revival post-flooding. Trends Plant Sci..

[85] Mittler R., Vanderauwera S., Gollery M., Van Breusegem F. (2004). Reactive oxygen gene network of plants. Trends Plant Sci..

[86] Hartman S., Sasidharan R., Voesenek L.A.C.J. (2021). The role of ethylene in metabolic acclimations to low oxygen. New Phytol..

[87] Van Der Straeten D., Zhou Z., Prinsen E., Van Onckelen H.A., Van Montagu M.C. (2001). A comparative molecular-physiological study of submergence response in lowland and deepwater rice. Plant Physiol..

[88] Yamauchi T., Shiono K., Nagano M., Fukazawa A., Ando M., Takamure I., Mori H., Nishizawa N.K., Kawai-Yamada M., Tsutsumi N. (2015). Ethylene biosynthesis is promoted by very-long-chain fatty acids during lysigenous aerenchyma formation in rice roots. Plant Physiol..

[89] Waadt R., Seller C.A., Hsu P.K., Takahashi Y., Munemasa S., Schroeder J.I. (2022). Plant hormone regulation of abiotic stress responses. Nat. Rev. Mol. Cell Biol..

[90] Schmitz A.J., Folsom J.J., Jikamaru Y., Ronald P., Walia H. (2013). *SUB1A*-mediated submergence tolerance response in rice involves differential regulation of the brassinosteroid pathway. New Phytol..

[91] Alpuerto J.B., Fukuda M., Li S., Hussain R.M.F., Sakane K., Fukao T. (2022). The submergence tolerance regulator *SUB1A* differentially coordinates molecular adaptation to submergence in mature and growing leaves of rice (*Oryza sativa* L.). Plant J..

[92] Nghi K.N., Tagliani A., Mariotti L., Weits D.A., Perata P., Pucciariello C. (2021). Auxin is required for the long coleoptile trait in *japonica* rice under submergence. New Phytol..

[93] Hoffmann-Benning S., Kende H. (1992). On the role of abscisic acid and gibberellin in the regulation of growth in rice. Plant Physiol..

[94] Minami A., Yano K., Gamuyao R., Nagai K., Kuroha T., Ayano M., Nakamori M., Koike M., Kondo Y., Niimi Y. (2018). Time-course transcriptomics analysis reveals key responses of submerged deepwater rice to flooding. Plant Physiol..

[95] Kurotani K., Hayashi K., Hatanaka S., Toda Y., Ogawa D., Ichikawa H., Ishimaru Y., Tashita R., Suzuki T., Ueda M. (2015). Elevated levels of CYP94 family gene expression alleviate the jasmonate response and enhance salt tolerance in rice. Plant Cell Physiol..

[96] Fukao T., Bailey-Serres J. (2008). Submergence tolerance conferred by *Sub1A* is mediated by SLR1 and SLRL1 restriction of gibberellin responses in rice. Proc. Natl. Acad. Sci. USA.

[97] Saika H., Okamoto M., Miyoshi K., Kushiro T., Shinoda S., Jikumaru Y., Fujimoto M., Arikawa T., Takahashi H., Ando M. (2007). Ethylene promotes submergence-induced expression of *OsABA8ox1*, a gene that encodes ABA 8′-hydroxylase in rice. Plant Cell Physiol..

[98] Yang S.H., Choi D. (2006). Characterization of genes encoding ABA 8′-hydroxylase in ethylene-induced stem growth of deepwater rice (*Oryza sativa* L.). Biochem. Biophys. Res. Commun..

[99] Chen D., Hou L., Qiu Z., Xu Q., Wang Q., Li M., Hao Z., Dong P., Zhang G., Hu J. (2026). Prime editing of an RR26-responsive *cis*-element enhances *DAHPS2* expression and submergence tolerance in rice through the shikimate pathway. Plant Commun..

[100] Fukao T., Bailey-Serres J. (2004). Plant responses to hypoxia-is survival a balancing act?. Trends Plant Sci..

[101] van Veen H., Triozzi P.M., Loreti E. (2024). Metabolic strategies in hypoxic plants. Plant Physiol..

[102] Verslues P.E., Bailey-Serres J., Brodersen C., Buckley T.N., Conti L., Christmann A., Dinneny J.R., Grill E., Hayes S., Heckman R.W. (2023). Burning questions for a warming and changing world: 15 unknowns in plant abiotic stress. Plant Cell.

[103] Perata P., Voesenek L.A. (2007). Submergence tolerance in rice requires *Sub1A*, an ethylene-response-factor-like gene. Trends Plant Sci..

[104] Xu Z., Wang Z., Jin N., Zheng Y., Shen Q., Ye L., Zhang G. (2026). Molecular and hormonal regulation of plant responses to waterlogging stress: From fundamental mechanisms to potential strategies for engineering crop tolerance. Plant Commun..

[105] Ashikari M., Nagai K., Bailey-Serres J. (2025). Surviving floods: Escape and quiescence strategies of rice coping with submergence. Plant Physiol..

[106] Jung K.H., Seo Y.S., Walia H., Cao P., Fukao T., Canlas P.E., Amonpant F., Bailey-Serres J., Ronald P.C. (2010). The submergence tolerance regulator *Sub1A* mediates stress-responsive expression of *AP2*/*ERF* transcription factors. Plant Physiol..

[107] Singh P., Sinha A.K. (2016). A positive feedback loop governed by SUB1A1 interaction with MITOGEN-ACTIVATED PROTEIN KINASE3 imparts submergence tolerance in rice. Plant Cell.

[108] Lin C.C., Chao Y.T., Chen W.C., Ho H.Y., Chou M.Y., Li Y.R., Wu Y.L., Yang H.A., Hsieh H., Lin C.S. (2019). Regulatory cascade involving transcriptional and N-end rule pathways in rice under submergence. Proc. Natl. Acad. Sci. USA.

[109] Nagai K., Ashikari M. (2023). Molecular mechanism of internode elongation in rice. Breed. Sci..

[110] Kuroha T., Nagai K., Gamuyao R., Wang D.R., Furuta T., Nakamori M., Kitaoka T., Adachi K., Minami A., Mori Y. (2018). Ethylene-gibberellin signaling underlies adaptation of rice to periodic flooding. Science.

[111] Nagai K., Mori Y., Ishikawa S., Furuta T., Gamuyao R., Niimi Y., Hobo T., Fukuda M., Kojima M., Takebayashi Y. (2020). Antagonistic regulation of the gibberellic acid response during stem growth in rice. Nature.

[112] Gómez-Ariza J., Brambilla V., Vicentini G., Landini M., Cerise M., Carrera E., Shrestha R., Chiozzotto R., Galbiati F., Caporali E. (2019). A transcription factor coordinating internode elongation and photoperiodic signals in rice. Nat. Plants.

[113] Sauter M., Rzewuski G., Marwedel T., Lorbiecke R. (2002). The novel ethylene-regulated gene *OsUsp1* from rice encodes a member of a plant protein family related to prokaryotic universal stress proteins. J. Exp. Bot..

[114] Hwang S.T., Choi D. (2016). A novel rice protein family of OsHIGDs may be involved in early signalling of hypoxia-promoted stem growth in deepwater rice. Plant Cell Rep..

[115] Lee K.W., Chen P.W., Lu C.A., Chen S., Ho T.H., Yu S.M. (2009). Coordinated responses to oxygen and sugar deficiency allow rice seedlings to tolerate flooding. Sci. Signal..

[116] Pucciariello C. (2020). Molecular mechanisms supporting rice germination and coleoptile elongation under low oxygen. Plants.

[117] Lu C.A., Ho T.H., Ho S.L., Yu S.M. (2002). Three novel MYB proteins with one DNA binding repeat mediate sugar and hormone regulation of alpha-amylase gene expression. Plant Cell.

[118] Lu C.A., Lin C.C., Lee K.W., Chen J.L., Huang L.F., Ho S.L., Liu H.J., Hsing Y.I., Yu S.M. (2007). The SnRK1A protein kinase plays a key role in sugar signaling during germination and seedling growth of rice. Plant Cell.

[119] Ye N.H., Wang F.Z., Shi L., Chen M.X., Cao Y.Y., Zhu F.Y., Wu Y.Z., Xie L.J., Liu T.Y., Su Z.Z. (2018). Natural variation in the promoter of rice calcineurin B-like protein10 (OsCBL10) affects flooding tolerance during seed germination among rice subspecies. Plant J..

[120] Kretzschmar T., Pelayo M.A.F., Trijatmiko K.R., Gabunada L.F.M., Alam R., Jimenez R., Mendioro M.S., Slamet-Loedin I.H., Sreenivasulu N., Bailey-Serres J. (2015). A trehalose-6-phosphate phosphatase enhances anaerobic germination tolerance in rice. Nat. Plants.

[121] Takashima T., Azumahara H., Hirano H., Hirata S., Fukuda M., Lamsal S., Miura K., Iwasaki Y., Fukao T. (2025). The heterotrimeric G protein γ3 subunit, *RGG3*/*GS3*, integrates sugar-starvation and hormone-responsive signaling pathways to promote coleoptile elongation during anaerobic germination in rice. Plant Mol. Biol..

[122] Nguyen C.D., Lu C.H., Chen Y.S., Lee H.T., Lo S.F., Wei A.C., Ho T.D., Yu S.M. (2025). Mitochondrial AOX1a and an H_2_O_2_ feed-forward signalling loop regulate flooding tolerance in rice. Plant Biotechnol. J..

[123] Sun J., Zhang G., Cui Z., Kong X., Yu X., Gui R., Han Y., Li Z., Lang H., Hua Y. (2022). Regain flood adaptation in rice through a 14-3-3 protein OsGF14h. Nat. Commun..

[124] Liang S., Xiong W., Yin C., Xie X., Jin Y.J., Zhang S., Yang B., Ye G., Chen S., Luan W.J. (2019). Overexpression of *OsARD1* improves submergence, drought, and salt tolerances of seedling through the enhancement of ethylene synthesis in rice. Front. Plant Sci..

[125] He Y., Sun S., Zhao J., Huang Z., Peng L., Huang C., Tang Z., Huang Q., Wang Z. (2023). UDP-glucosyltransferase OsUGT75A promotes submergence tolerance during rice seed germination. Nat. Commun..

[126] Lu X., Yang L., Shen L., Zhan C., Dai L., Huang L., Zhang Q., Fang Y., Ren D., Zhu L. (2025). Genome-wide association study uncovers a novel gene responsible for rice seedling submergence tolerance. Plant Biotechnol. J..

[127] Guo F., Han N., Xie Y., Fang K., Yang Y., Zhu M., Wang J., Bian H. (2016). The miR393a/target module regulates seed germination and seedling establishment under submergence in rice (*Oryza sativa* L.). Plant Cell Environ..

[128] Liu S., Sun Z., Lv L., Huang X., Fan H., Li M., Shi B., Gao Y., Ai H., Xu D. (2025). Transcriptome analysis of submergence stress in rice provides insights into the molecular mechanism of rice response to flooding and the roles of *OsEXPB3* under submergence. Agriculture.

[129] Mittal L., Tayyeba S., Sinha A.K. (2022). Finding a breather for *Oryza sativa*: Understanding hormone signalling pathways involved in rice plants to submergence stress. Plant Cell Environ..

[130] Fukao T., Yeung E., Bailey-Serres J. (2012). The submergence tolerance gene *SUB1A* delays leaf senescence under prolonged darkness through hormonal regulation in rice. Plant Physiol..

[131] Fukao T., Yeung E., Bailey-Serres J. (2011). The submergence tolerance regulator SUB1A mediates crosstalk between submergence and drought tolerance in rice. Plant Cell.

[132] Neeraja C.N., Maghirang-Rodriguez R., Pamplona A., Heuer S., Collard B.C., Septiningsih E.M., Vergara G., Sanchez D., Xu K., Ismail A.M. (2007). A marker-assisted backcross approach for developing submergence-tolerant rice cultivars. Theor. Appl. Genet..

[133] Mao C., Wang S., Jia Q., Wu P. (2006). *OsEIL1*, a rice homolog of the Arabidopsis *EIN3* regulates the ethylene response as a positive component. Plant Mol. Biol..

[134] Kende H., van der Knaap E., Cho H.T. (1998). Deepwater rice: A model plant to study stem elongation. Plant Physiol..

[135] Nagai K., Kurokawa Y., Mori Y., Minami A., Reuscher S., Wu J., Matsumoto T., Ashikari M. (2022). *SNORKEL* genes relating to flood tolerance were pseudogenized in normal cultivated rice. Plants.

[136] Kawano R., Doi K., Yasui H., Mochizuki T. (2008). Mapping of QTLs for floating ability in rice. Breed. Sci..

[137] Sasaki A., Ashikari M., Ueguchi-Tanaka M., Itoh H., Nishimura A., Swapan D., Ishiyama K., Saito T., Kobayashi M., Khush G.S. (2002). Green revolution: A mutant gibberellin-synthesis gene in rice. Nature.

[138] Spielmeyer W., Ellis M.H., Chandler P.M. (2002). *Semidwarf* (*sd-1*), “green revolution” rice, contains a defective gibberellin 20-oxidase gene. Proc. Natl. Acad. Sci. USA.

[139] Monna L., Kitazawa N., Yoshino R., Suzuki J., Masuda H., Maehara Y., Tanji M., Sato M., Nasu S., Minobe Y. (2002). Positional cloning of rice semidwarfing gene, *sd-1*: Rice “green revolution gene” encodes a mutant enzyme involved in gibberellin synthesis. DNA Res..

[140] Singh A., Septiningsih E.M., Balyan H.S., Singh N.K., Rai V. (2017). Genetics, physiological mechanisms and breeding of flood-tolerant rice (*Oryza sativa* L.). Plant Cell Physiol..

[141] Ho V.T., Tran A.N., Cardarelli F., Perata P., Pucciariello C. (2017). A calcineurin B-like protein participates in low oxygen signalling in rice. Funct. Plant Biol..

[142] Anumalla M., Khanna A., Catolos M., Ramos J., Sta. Cruz M.T., Venkateshwarlu C., Konijerla J., Pradhan S.K., Dash S.K., Das Y. (2025). Future flooding tolerant rice germplasm: Resilience afforded beyond *Sub1A* gene. Plant Genome.

[143] Septiningsih E.M., Pamplona A.M., Sanchez D.L., Neeraja C.N., Vergara G.V., Heuer S., Ismail A.M., Mackill D.J. (2009). Development of submergence-tolerant rice cultivars: The *Sub1* locus and beyond. Ann. Bot..

[144] Toledo A.M.U., Ignacio J.C.I., Casal C., Gonzaga Z.J., Mendioro M.S., Septiningsih E.M. (2015). Development of improved Ciherang-Sub1 having tolerance to anaerobic germination conditions. Plant Breed. Biotech..

